# Supplementary material for: MINFLUX microscopy resolves subunits of the cardiac ryanodine receptor and its 3D orientation in cells
Source: Nat Commun. 2025 Dec 21;17:1044. doi: 10.1038/s41467-025-67801-6 (PMC12847994; doi:10.1038/s41467-025-67801-6)
Supplement: Supplementary file 1 — Supplementary Information [file 41467_2025_67801_MOESM1_ESM.pdf]

MINFLUX microscopy resolves subunits of the cardiac ryanodine receptor and its 3D orientation in cells

Alexander H Clowsley[1*], Anna Meletiou[1*], Radoslav Janicek[1], Alexandre F. E. Bokhobza[1], Evelina Lučinskaitė[1], Gabriela Bleuer[1], Isabelle Jansen[2], Peter P. Jones[3], William E. Louch[4,5], Christian Soeller[1, Δ]

Affiliations

[1]Department of Physiology, University of Bern, Bern, Switzerland.
[2] Abberior Instruments GmbH, Göttingen, Germany.
[3]Department of Physiology, School of Biomedical Sciences and HeartOtago, University of Otago, Dunedin, New Zealand.
[4]Institute for Experimental Medical Research, Oslo University Hospital and University of Oslo, Oslo, Norway.
[5]K.G. Jebsen Centre for Cardiac Research, University of Oslo, Oslo, Norway.
*These authors contributed equally.
Δ Corresponding author: christian.soeller@unibe.ch

Supplementary Methods and supplementary Figures

Supplementary Methods

Analysis of 3D MINFLUX and SMLM data

For data analysis and visualization, raw MINFLUX localization data were exported using Abberior Instruments Imspector software[1] (MINFLUX version) to Numerical Python (Numpy) NPY format. The NPY formatted data was directly imported into PYMEVisualize[2] using custom plugin code in the `PYMEcs.IO.MINFLUX` module of the PYME-extra repository (available on github at github.com/csoeller/PYME-extra). Upon importing the data a foreshortening factor of 0.72 was applied to scale all z-coordinates, similar as described previously[3], and registered in the metadata. This factor accounts for refractive index mismatch between the coverslip glass and cell material and was calibrated by measuring the NPC ring spacing in 3D MINFLUX data from U2OS-Nup96-mEGFP cells to give a mean of ~50 nm after foreshortening correction.

SMLM data was axially calibrated as described[4] using nanopillars which determined an effective foreshortening of 0.64. This correction includes distortions from refractive index mismatch and any scaling corrections of the nominal focusing piezo movement. The foreshortening was implemented with a module from the PYME-extra package that scaled the z coordinates by the determined correction factor as part of the a PYME localization data processing pipeline[2].

Estimating docking strand and RyR2 subunit locations

Raw MINFLUX localizations were processed by combining localizations to represent different aspects of the underlying structures. During attachment to the docking strand on sdABs an emitting imager strand is typically localized several times in direct succession by rapidly repeating MINFLUX localization iterations, until the imager strand eventually unbinds. These successive localizations are assigned to the same trace via the same trace ID (exported parameter TID)[5]. Localization precision was estimated as the standard deviation of MINFLUX localization coordinates of localizations belonging to the same TID[3,5], limiting localizations to traces that contain at least 4 localizations. We also generally applied filtering by the center-frequency ratio (CFR)[1,5], limiting further analysis to raw MINFLUX localizations with CFR ≤ 0.75.

Docking strand locations were obtained from raw MINFLUX localizations by combining all localizations from the same trace (TID) to obtain a single localization that represents the location of

the docking strand on the sdAB marker. The resulting docking strand locations have a higher localization precision and each such location is calculated as the centroid of the raw MINFLUX localizations belonging to its trace [5,6].

Subunit locations (molecule locations). To calculate estimates of unique subunit locations when imaging RyR2s with FP insertions in their subunits (i.e. $RyR2_{D4365\text{-}GFP}$ and $RyR2_{T1365\text{-}PATagRFP}$) we used an approach similar to the procedure used previously to reassign filtered MINFLUX localizations to individual molecules[6]. In this three-step procedure we first combine raw MINFLUX localizations to obtain docking strand locations as described above. In a second step a DBSCAN clustering of docking strand locations is performed to group locations that arise from a potentially repeatedly localized docking strand of an sdAB. The distance ε was chosen to be small enough to avoid incorrectly combining dye locations from sdABs on different subunits. Given that docking strand locations also have a localization error, typically < 2 nm, a DBSCAN ε = 1 nm was adopted. In a final step, DBSCAN clustered docking strand locations from the same DBSCAN cluster are combined into a single subunit location estimate. The location is calculated as the weighted centroid of the docking strand locations in the same DBSCAN cluster. This was implemented via the `MergeClumps` module in PYME which combines localization coordinates weighted by localization error.

Implementation. Filtering, grouping and combining of localizations were carried out using a PYME localization data processing pipeline with operations represented by recipes[2] that comprise a combination of localization data processing modules to implement operations such as DBSCAN clustering, combining (merging) localizations, filtering, etc as previously described[2].

Combining localizations. When combining N localizations with coordinates x_i, $i = 1..N$ and corresponding localization precisions σ_i, a coordinate x_c of the combined location is calculated as the weighted centroid according to

$$x_c = \sum_{i=1}^{N} w_i\, x_i \quad (1) \quad \text{where}$$

$$w_i = \frac{1}{w}\frac{1}{\sigma_i^2} \quad (2) \text{ and}$$

$$w = \sum_{i=1}^{N} \frac{1}{\sigma_i^2} \quad (3)$$

The localization error σ_c of the combined localization coordinate x_c is

$$\sigma_c = \sqrt{\frac{1}{w}} \quad (4) \qquad \text{and therefore one can also write.} \qquad w_i = \frac{\sigma_c^2}{\sigma_i^2} \quad (5).$$

If all localizations to be combined have the same localization error $\sigma_i = \sigma_0$, $i = 1..N$, e.g. when combining the raw MINFLUX localizations belonging to the same trace (TID), this simplifies to

$$x_c = \frac{1}{N}\sum_{i=1}^{N} x_i \quad (6) \quad \text{and}$$

$$\sigma_c = \frac{\sigma_0}{\sqrt{N}} \quad (7)$$

This localization combining approach is implemented in the `MergeClump` module of `PYMEVisualize`. The calculations are applied to all 3D coordinate axes, i.e. the x_i and corresponding σ_i respectively represent the x, y and z coordinates which are all processed in this way.

Visualizing 3D MINFLUX data

MINFLUX localizations were visualized in the `PYMEvisualize` point viewer with localizations shown as OpenGL based Gaussian shaped pointsprites[2] and colored according to one of several localization properties including z location, acquisition time, cluster size or cluster ID (a unique number given to each cluster) which results in a Gaussian rendering in 3D. In some 3D visualizations localizations were rendered as 3D spheres using the `PYMEvisualize` point viewer.

To determine image resolution we calculated Fourier Ring Correlation (FRC)[7,8] or Fourier Shell Correlation (FSC) measures. To calculate the FRC or FSC values, raw MINFLUX localizations were grouped into two groups of localizations using the “Split by Time Blocks for FRC” functionality of `PYMEVisualize` using a Time Block size of 10. For FRC measurements the two groups of localizations were rendered into two 2D images using the Gaussian rendering method of `PYMEVisualize` and a pixel size of 3 nm. FRC resolution was determined as the 1/7 threshold frequency inverse using the FRC module of PYME-extra. For FSC calculations, the two groups of localizations were rendered into two 3D volume images using the Gaussian rendering method of `PYMEVisualize` and a pixel size of 3 nm. The volume images were saved in MRC format using the Python package `mrcfile` which provides a Python implementation of the MRC2014 file format. The data was uploaded to the EMDB FSC server at https://www.ebi.ac.uk/emdb/validation/fsc which calculated the FSC curve from the provided data. FSC resolution was determined as the 1/7 threshold spatial frequency inverse.

Estimates of effective labeling efficiency from MINFLUX 3D NPC data

To estimate effective labeling efficiency an approach similar to the strategy introduced by Thevathasan et al.[9] was used, but fully extended to 3D. MINFLUX 3D data of U2OS-Nup96-mEGFP labeled with anti-GFP sdABs modified with DNA-PAINT docking strands were first rendered to a 2D image using Gaussian rendering in PYMEvisualize (Supplementary Fig. 4 A) and locations of NPCs were detected by a semi-manual approach in Fiji[10] and stored as regions-of-interest (ROIs) in ImageJ ROI format. ROIs were read into the PYMEvisualize[2] SMLM data visualizer using the Python roifile package and utilized to generate a mask and label 3D MINFLUX localizations belonging to detected NPCs with a unique ID. For each NPC the associated set of localizations (identified by a unique ID label) were fit to a 3D double ring template using a maximum-likelihood algorithm similar to an approach described previously[11]. A 3D template was constructed from two rings (nominal diameter 107 nm) spaced 50 nm apart in z and convolved with a Gaussian (sigma = 5 nm) in 3D to provide a smooth template for alignment (Supplementary Fig. 4 B). A small evenly distributed probability was added to account for background localizations[11]. We chose a smooth model versus a detailed NPC template for robust parameter estimation[11], sufficient for robust estimates of labeled versus unlabeled segments of the NPC data sets. Similarly to previous work, for further robustness we do not rely on resolving the pairs of nearby Nup96 sites (laterally ~12 nm apart) in the NPC structure but rather test for labeling of each of 8 radial segments in the NPC structure that each contain the two nearby Nup96 sites[9], and do this separately for cytoplasmic and nucleoplasmic parts of the NPC. Prior to template fitting the MINFLUX localizations are typically slightly shifted relative to the template and also exhibit a visible tilt angle (Supplementary Fig. 4 B, top row).

The optimization algorithm works on the negative logarithm of the likelihood (NLL) function, i.e. the maximum likelihood is found my minimizing the NLL. The NLL for the given set of localizations and current transformation parameters is obtained by evaluating the NLL template function at each transformed localization coordinate and summing the contribution from all localizations that belong to the NPC[11]. To minimize the NLL, 7 parameters are varied which describe coordinate transformations to line up the MINFLUX data with the template: 3 spatial coordinates to define the NPC center, 2 angles in Euler notation describing the rotation of the NPC central axis and 2 scaling factors to account for small variations in ring diameter and inter-ring spacing, respectively. Minimization is performed with a global optimizer to avoid becoming trapped in local minima[11]. In

our implementation we used the `basinhopping` function of the `scipy.optimize` package which performs basin-hopping, a two-phase method that combines a global stepping algorithm with local minimization at each step[12]. The `basinhopping` function was used with the L-BFGS-B method, i.e. the limited-memory BFGS algorithm that approximates the Broyden–Fletcher–Goldfarb–Shanno algorithm (BFGS), extended with simple bound constraints on variables[13].

The transformed coordinates that minimize the NLL are then used for further determination of labeling of NPC segments in the two rings. An example of aligned localizations is shown in Supplementary Fig. 4 B, bottom row. In addition, the optimal fitting parameters are also used to display the transformed template overlaid with the fitted MINFLUX localizations in PYMEvisualize, see Supplementary Fig. 4 C,D. Notably, the central symmetry axis is also displayed in these overlays and it exhibits visible variation in NPC angles across the field of view.

In the next step, rotational alignment of the aligned NPC data is performed as described for 2D data[9] but in our implementation separately for the cytoplasmic and nucleoplasmic rings as illustrated in Supplementary Fig. 4 E. To determine the segment boundaries for an NPC data set we calculate a metric that looks at angles of events and assigns the smallest penalty (zero) at the center of a π/4 (45 degree) segment and increases linearly towards 0.5 at the edges of segments. Contributions from all NPC events are summed up to determine a penalty measure. Segment boundaries are rotated in a range of 0 to π/4 and the rotation angle that minimizes the total penalty is the rotation that defines the segment borders for the following determination of labeled segments. The number of labeled segments in the 8-fold symmetry of the NPC structure is determined using a threshold value. In our analysis, a threshold of 1 localization was used due to the high signal-to-noise in the data and a threshold analysis that confirmed its suitability (shown in Supplementary Fig. 4 H). Each NPC can exhibit from 0 to 16 labeled segments, as the labeling evaluation is carried out separately for cytoplasmic and nucleoplasmic rings.

The data from all NPCs in a dataset evaluated in this way were pooled into a cumulative histogram and fitted to a probabilistic model that predicts the relative number of 0 to 16 labeled segments as a function of the fit parameter p_{LE} which denotes the labeling efficiency. The model is obtained by starting from the binomial probability distribution of having exactly *k* successes when attempting to label *n* independent sites with the same labeling probability p_{LE}

$$f(k, n, p_{\mathrm{LE}}) = \binom{n}{k} p_{\mathrm{LE}}^{k} (1 - p_{\mathrm{LE}})^{n-k} \qquad (8)$$

Similarly to previous work, we test for labeling of each segment in the 8-fold symmetry NPC structure with each segment containing two nearby Nup96 sites[9]. Accordingly, the probability $p_{\mathrm{seg,unlabeled}}$ that a segment of the NPC structure is unlabeled is obtained from the binomial probability that both Nup96 sites are not labeled

$$p_{\mathrm{seg,unlabeled}} = f(0{,}2, p_{\mathrm{LE}}) \quad (9)$$

which gives the probability $p_{\mathrm{seg,labeled}}$ that at least one of the sites is labeled as

$$p_{\mathrm{seg,labeled}} = 1 - p_{\mathrm{seg,unlabeled}} = 1 - f(0{,}2, p_{\mathrm{LE}}) \quad (10)$$

From this expression we obtain the probability $p(N, p_{\mathrm{LE}})$ that *N* out of 16 segments are labeled as

$$p(N, p_{\mathrm{LE}}) = f\big(N, 16, p_{\mathrm{seg,labeled}}\big) = f\big(N, 16{,}1 - f(0{,}2, p_{\mathrm{LE}})\big) \quad (11)$$

and similarly, the cumulative probability $p_c(N, p_{\mathrm{LE}})$ which we show in plots and use as fitting function, as

$$p_c(N, p_{\mathrm{LE}}) = \sum_{k=0}^{N} p(k, p_{\mathrm{LE}}) \quad (12)$$

Processing both experimental and model histograms in cumulative form includes the advantage that the curves have a characteristic sigmoidal shape that shifts to the right with increasing p_{LE}, aiding convenient visual inspection of effective labeling performance, see Supplementary Fig. 4 F. The best fit parameter p_{LE} together with its uncertainty Δp_{LE} (obtained from the square root of the relevant diagonal entry of the covariance matrix) are then provided as estimate of effective labeling efficiency.

Suitability of a threshold for deciding labeling of a segment was tested using background data from a region distal from the immediate vicinity of the nuclear envelope. As shown in Supplementary Fig. 4 G, using a nucleoplasmic region above the level of the NPCs (Background region in Supplementary Fig. 4 Gii) p_{LE} was determined first just using the actual NPC localizations and then also (case 1) only using the background region with the same NPC mask obtained from the actual NPC data and (case 2) displacing all the localizations in the background region into the plane of the NPCs (by reducing the z-coordinates of localizations in the background region by 220 nm to move them into the NPC region) and thus determination of p_{LE} in the presence of the additional background. This analysis was carried out for 3 typical datasets that exhibit a p_{LE} of 59.1 % (N = 3 datasets from 2 independent realizations, Supplementary Fig. 4 Hi) as reference value. Just evaluating the data from the background region alone (case 1) gives an apparent labeling efficiency LE_{BG} of 3.0 % and, more importantly, in the additional presence of the background (case 2), the mean labeling efficiency only increases from the reference value 59.1 % to 59.3 %, i.e. a difference ΔLE of 0.2% resulting from additional background (N = 3 datasets from 2 independent realizations, Supplementary Fig. 4 Hii). This very small increase due to background localizations shows that the NPC data is highly specific and that a threshold of one compound localization (obtained from coalescing a trace of raw MINFLUX localizations as described above) is suitable for determining segment labeling. Overall, it suggests that estimated labeling efficiencies are perturbed by background localizations by one percent at most, most likely considerably less.

The whole procedure is implemented in the `PYMEcs.Analysis.NPC` module of the `PYME-extra` package, specifically in the `LLmaximizerNPC3D`, `NPC3D` and `NPC3DSet` classes. A graphical user interface is provided by the `NPCcalcLM` module as a plugin for PYMEVisualize.

NPC geometry analysis

NPC geometries from MINFLUX 3D imaging and SMLM biplane 3D imaging of Nup96-EGFP labeled with DNA-PAINT sdABs against GFP were analyzed and compared. As described in the previous section Nup96-EGFP distribution was analyzed using maximum-likelihood fitting of double-ring templates. The fitting involves scaling of ring diameter and ring spacing to obtain best fit agreement between the template and the distribution of localizations. In this way, measures of ring diameter, ring spacing and average NPC 3D images were obtained using MINFLUX 3D imaging and SMLM biplane 3D imaging, respectively, and compared as shown in Supplementary Fig. 5. Prior to maximum likelihood fitting SMLM biplane data was filtered to limit it to combined localizations with an axial localization precision of 13.5 nm or better. To obtain averaged 3D images of Nup96-EGFP distribution, data from many NPCs were fit, aligned along the z axis and normalized to a standard ring diameter and spacing (here 110 and 50 nm, respectively) as described previously in similar studies[11,14]. Analysis was restricted to NPCs with high goodness of fit to the template, measured by dividing the magnitude of the best fit NLL by the number of events. The resulting overlaid localization events were 3D Gaussian rendered using Gaussian widths of 1 nm and mean-projected along the z axis (using only data from the nucleoplasmic side) or along the y axis to produce x-y and x-z projections, respectively. Visual comparison of MINFLUX imaged and aligned NPCs versus SMLM

biplane imaged and aligned NPCs suggests that MINFLUX images are higher resolved; especially the axial resolution of the two rings is by comparison less clear in the 3D SMLM data (Supplementary Fig. 5A, B). Typically, ring diameter estimates in MINFLUX and SMLM data cluster closely around the mean value (Supplementary Fig. 5C, D) whereas the ring spacing estimates from SMLM data are more variable (see example in Supplementary Fig. 5D – 6.8 nm) than estimates from MINFLUX data (see example in Supplementary Fig. 5C – 2.7 nm), presumably reflecting the improved axial resolution in 3D MINFLUX data. This is broadly maintained when comparing the variability of means across data sets (Supplementary Fig. 5E), where MINFLUX derived mean ring diameter is r = 111.9 ± 2.2 nm (mean ± standard deviation, N = 5 MINFLUX datasets from n = 2 independent realizations, 365 NPCs in total) versus SMLM mean ring diameter r = 117.9 ± 4.1 nm (mean ± standard deviation, N = 4 SMLM datasets from n = 2 independent realizations, 228 NPCs in total), i.e. approximately twice as large a standard deviation in SMLM derived data. Similarly, MINFLUX derived mean ring spacing is s = 49.6 ± 1.9 nm (mean ± standard deviation, N = 5 MINFLUX datasets from n = 2 independent realizations, 365 NPCs in total) versus SMLM mean ring spacing s = 58.9 ± 3.9 nm (mean ± standard deviation, N = 4 SMLM datasets from n = 2 independent realizations, 228 NPCs in total). Ring diameters appear slightly larger in SMLM derived data, and the mean ring spacing is slightly larger in SMLM which could reflect, for example, residual uncorrected drift in SMLM data in conjunction with slightly lower resolution (note that drift in our MINFLUX data is corrected by the MINFLUX beam monitoring system). The MINFLUX derived data compares closely with previous studies which report a mean ring spacing close to 50 nm[9,11] (note that an average ring spacing of ~50 nm was used to calibrate the MINFLUX foreshortening value, see above) and also ring radii agree within the reported standard deviations[9,11] when our diameter measure is converted to a radius value, i.e. 55.9 ± 1.1 nm. In this context we note that small variations in ring diameter across studies may reflect the dynamic nature of NPC diameter which can respond to external stimuli[15] and could thus reflect subtle differences in sample preparation. The somewhat less well-defined geometry of Nup96-EGFP distribution in our SMLM data is also seen when averaged 3D images are compared (Supplementary Fig. 5F). In a MINFLUX based averaged image some of the ~12nm distal peaks resulting from adjacent Nup96 sites can be distinguished which are not discernible in the SMLM averaged image, similar as in previously shown averaged images[11]. It should be noted that specific SMLM implementations can achieve excellent axial resolution (e.g.[16,17]). Accordingly, the differences observed here may reflect the SMLM biplane implementation in our custom-built setup and results could vary with other SMLM implementations. In any case, the high precision achievable with 3D DNA-PAINT MINFLUX imaging is notable and the use of NPC imaging as a biological standard available across laboratories provides a useful benchmark for overall system performance.

RyR2 cluster analysis

RyR2 cluster analysis was performed using DBSCAN clustering of subunit locations, taking the aspect of MINFLUX data into account that DBSCAN clustering of subunit locations, not RyR2 locations per se, is conducted. A distance ε = 100 nm was used to define functional RyR2 clusters, as 100 nm is often taken as the diffusion distance over which Ca^{2+} release from a local RyR2 has a reasonable probability to trigger release from adjacent RyR2s[18,19]. The distance is thus justified based on the functional definition of a RyR2 cluster as a group of RyR2s that will likely jointly open during the time course of a Ca^{2+} spark.

RyR2 cluster sizes obtained by DBSCAN clustering with ε = 100 nm are reported in two ways, (a) as the number of labeled subunits in a cluster and (b) the number of RyR2s in the corresponding clusters using estimates of labeling efficiency p_{LE} for correction when available (primarily with HEK293 cells expressing RyR2-GFP). We obtain a corrected number nRyR of RyR2s in the cluster from the observed number of labeled subunit locations nSU in the cluster according to

$$\text{nRyR} = \frac{\text{nSU}}{4}\frac{1}{p_{\text{LE}}} \quad (13)$$

Deviation of cluster shapes from a plane

To illustrate how much RyR2 clusters in peripheral couplings deviate from a flat geometry we show a typical dataset in Supplementary Fig. 8 from several angles in 3D. In Supplementary Fig. 8 A-F it is apparent that clusters have a somewhat curved shape (akin to shallow cups), at the surface membrane. In addition, we show a typical cluster with its corresponding alpha shape[20,21] determined using the MATLAB alphaShape functionality. As a measure of the scattering of points, we calculated the standard deviation of z-coordinates (~27 nm) which was typically about 10 times larger than localization errors themselves (2-3 nm), indicating considerable deviation from a flat RyR2 arrangement, as shown in Supplementary Fig. 8 F & G.

Generation of simulated data from EM tomography data

Starting with RyR2 cluster morphologies as determined in ventricular myocytes using EM tomography[22,23] we generated simulated labeled subunit locations assuming variable degree of labeling. Briefly, RyR2 positions were drawn in Inkscape as squares to match the red squares in Fig. 1 from Asghari et al., 2014[22] and Fig. 2 from Asghari et al., 2020[23]. The coordinates of the resulting squares stored in Inkscape SVG format were read in using Python with the ElementTree XML API as implemented in xml.etree.ElementTree and further processed to define the locations where GFP/tagRFP residues are positioned[24,25] (see Supplementary Fig. 7). Based on these subunit label locations simulated MINFLUX localizations were generated by adding localization errors with a standard deviation of 3.5 nm. Various labeling efficiencies were simulated by randomly selecting a subset of these localizations with the fraction chosen according to the p_{LE} value that was to be simulated.

Assembly of molecular schematics

For marker size schematics and RyR2 labeling schematics, PDB structures were rendered using the Mol* Viewer[26] and assembled in Inkscape to scale, using structures with PDB IDs 12FX, 6JI8, 5SYF, 1IGT, 3M22, 4OGS, 6I2G.

Data availability and software

Processing was conducted using the `PYME` software package[2] with apps including `PYMEVisualize` and `PYMEImage` as well as by directly using `PYME` API functions in `Jupyter` notebooks (using `Python` 3) and the `PYME` plugin package `PYME-extra` (`https://github.com/csoeller/PYME-extra`). Versions of python packages `PYME-test-env`, `python-microscopy` and `PYME-extra` used in this study are available via Zenodo DOIs `https://doi.org/10.5281/zenodo.17768735`[27], `https://doi.org/10.5281/zenodo.17768283`[28] and `https://doi.org/10.5281/zenodo.17763621`[29], respectively. MINFLUX DNA-PAINT source files, `Jupyter` notebooks (`Python` notebooks), all source data and analysis scripts are available through figshare at the DOI `https://doi.org/10.6084/m9.figshare.23733579`[30]. Raw data is available in the related figshare repository at DOI `https://doi.org/10.6084/m9.figshare.29196023`[31].

Supplementary Figures

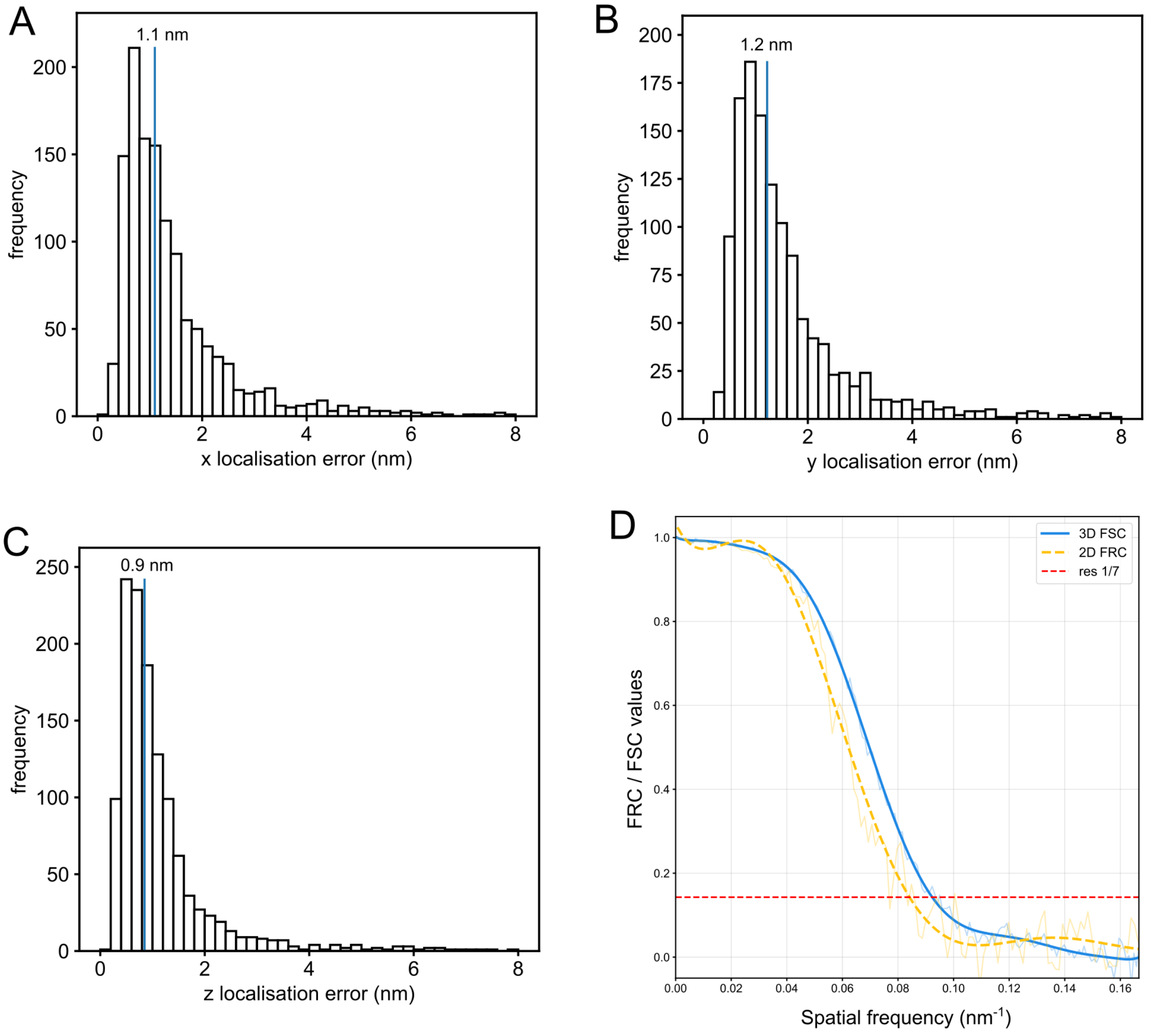

Supplementary Figure 1. Localization errors and resolution measures. **A-C**. Shown are histograms of x, y and z localization errors of docking strand locations that were obtained by combining raw MINFLUX localization as described in the Methods. Note that median localization errors are well below 2 nm in all directions. **D**. Fourier ring correlation (FRC) and Fourier shell correlation (FSC) of a cardiac myocyte $RyR2_{T1365\text{-}PATagRFP}$ MINFLUX dataset, calculated as detailed in the methods. The FRC measurement encompassing only the two-dimensional data yields a 2D resolution of 11.9 nm. The FSC which incorporates the same xy data as the FRC but also includes z coordinates after rendering into a volume data set measures a 3D resolution of 10.8 nm. The similarity between the FRC and FSC resolution values is a further demonstration of the near homogeneous localization of docking strands in all directions when using 3D MINFLUX DNA-PAINT. Source data are provided with this paper.

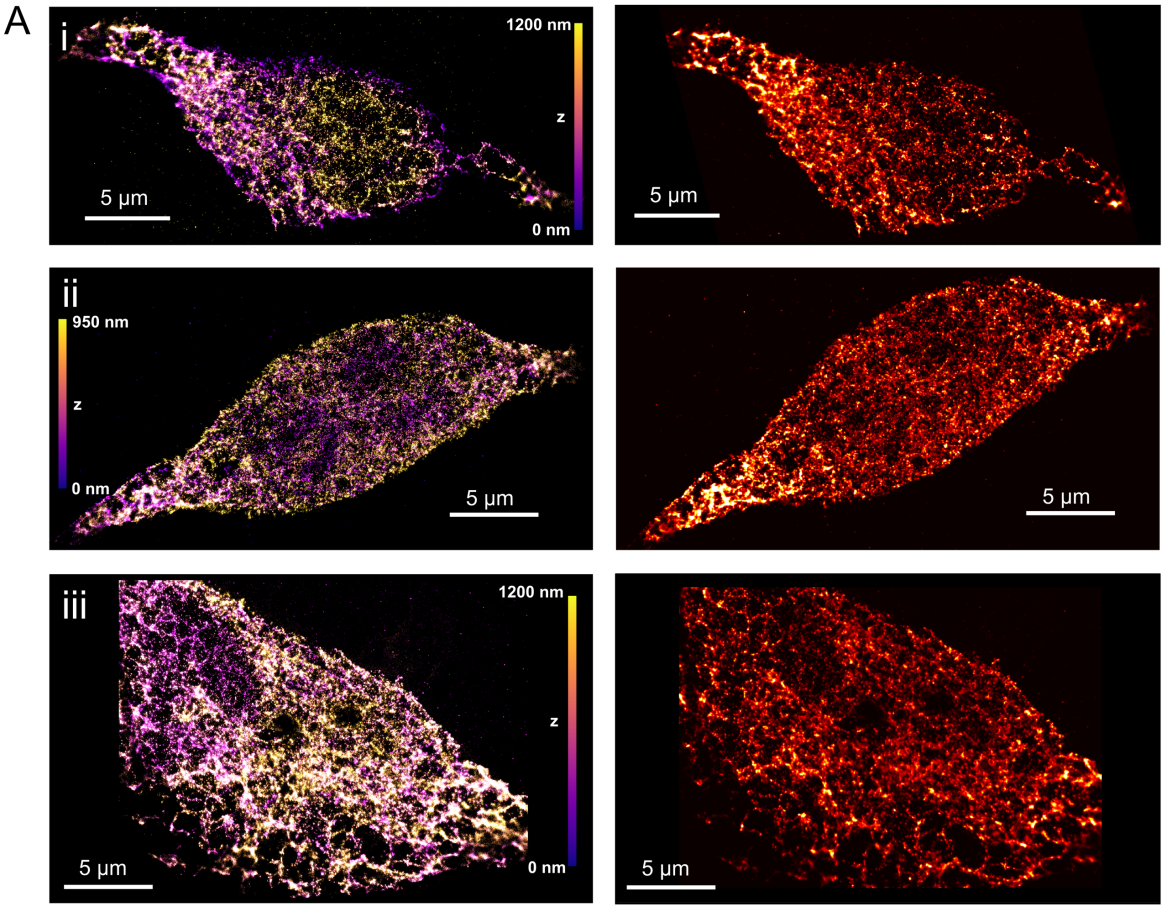

Supplementary Figure 2. Widefield DNA-PAINT data of HEK293 cells stably expressing $RyR2_{D4365\text{-}GFP}$. **A**. Whole cell overviews using 3D widefield DNA-PAINT super-resolution imaging with biplane axial localization. Left panels show OpenGL based PYMEVisualize 3D renderings with axial position color-coded, right panels show Gaussian 2D renderings of the corresponding panels on the left. N = 3 independent repeats with similar results.

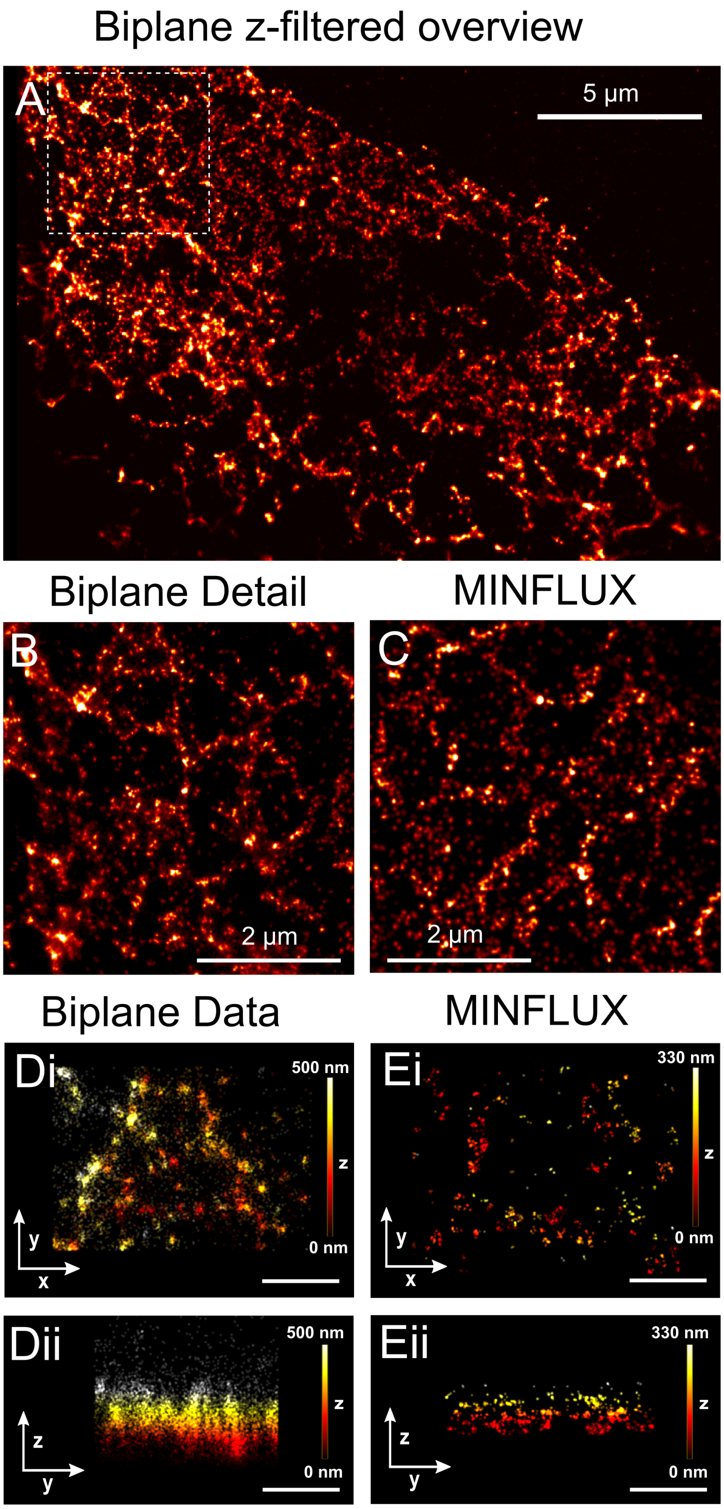

Supplementary Figure 3. Comparison between MINFLUX and widefield DNA-PAINT data. Part 2 – **A**. Overview of the data as in Supplementary Fig. 2 Aiii but filtered to only include localizations in an axial range of 500 nm to match the corresponding MINFLUX dataset. **B**. Magnified detail from A (indicated by box in A). **C**. An area of equivalent lateral extent from an MINFLUX DNA-PAINT dataset, rendered and blurred with a Gaussian of 15 nm diameter (standard deviation) to more closely match the lower resolution in the widefield DNA-PAINT dataset. Note the qualitatively similar appearance. **D** and **E**. Comparison of x-y (i) and x-z (ii) views from widefield biplane data (D) and MINFLUX 3D data (E). Note the much poorer z-detail in the widefield DNA-PAINT data. N = 3 independent repeats with similar results. Scale bars D, E: 500 nm.

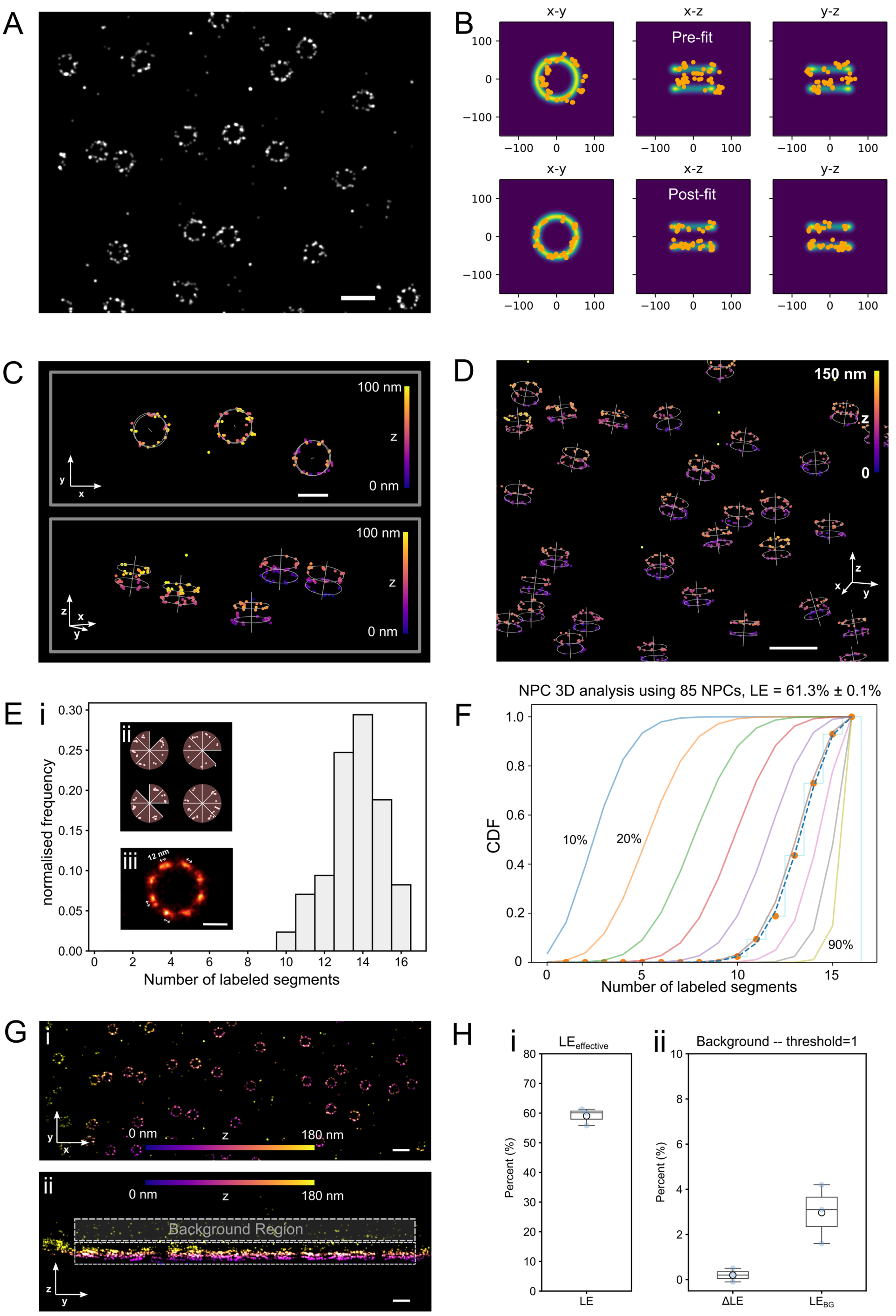

Supplementary Figure 4. Determination of effective labeling efficiency with NPC structures obtained from U2OS-Nup96-mEGFP labeling. **A**. A 2D rendering of the MINFLUX 3D data was rendered and processed in Fiji to detect NPC structures. **B**. Localizations belonging to identified NPC structures were fit using a maximum-likelihood approach implemented with a double ring template. The top row shows various views of the unaligned data from a single NPC prior to fitting and the bottom row indicates the best fit transformation aligning the data with the template. **C**. The best fit data was used to generate template overlays (double ring plus a line indicating the central symmetry axis through the pore). **D**. In this larger field-of-view, otherwise as in C, the variation in NPC axis alignment across the field is visible. **E**. Labeling of segments was determined to generate an experimental histogram (i) following rotational alignment of fitted data to segment boundaries (ii). The aligned NPC localizations can also be overlaid to produce and render an average distribution of Nup96 localizations (iii) which clearly exhibits the 8-fold symmetry and also shows ~12nm distal peaks resulting from the two Nup96 sites in a segment. **F**. Fitting of the cumulative experimental histogram (dots) to the model yields an estimate of effective labeling efficiency as best fit (dashed line), here 61.3 % ± 0.1 %. **G** and **H**. Testing of labeling threshold against background contribution as described in text, supporting the suitability of a threshold of 1. N = 3 independent replicates. Box plots show the median (center line), the 25th and 75th percentiles (bounds of the box), and the mean (large circular marker). Whiskers extend to the most extreme data points within 1.5x the interquartile range, and all individual data points are displayed as a swarm overlay. Scale bars A, D, G: 200nm, C: 100 nm, E: 50 nm. Source data are provided with this paper.

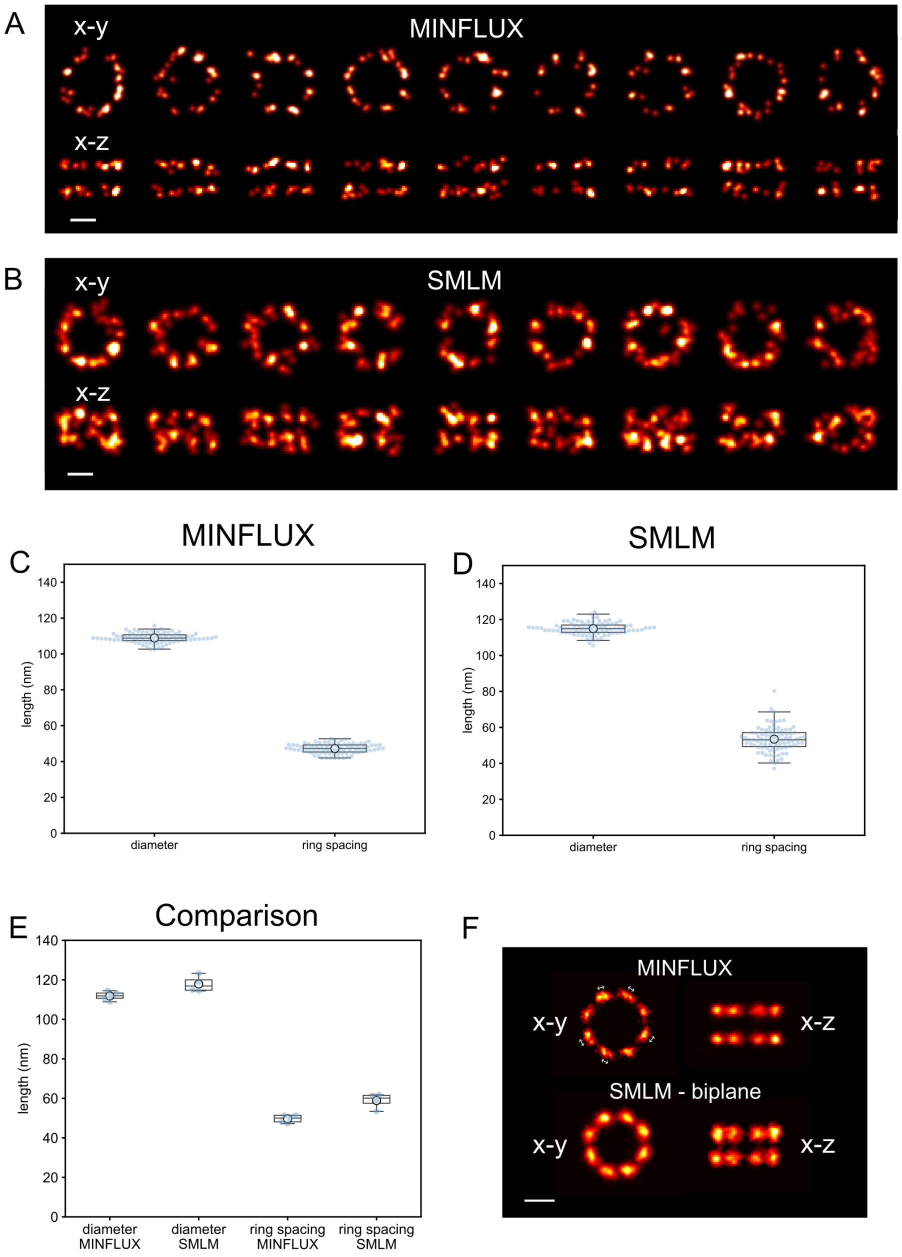

Supplementary Figure 5. Analysis of NPC geometries obtained with MINFLUX and SMLM 3D imaging. **A**. Gallery of Nup96-EGFP labeled NPCS from a MINFLUX DNA-PAINT dataset in lateral (x-y) and axial (x-z) views. **B**. Equivalent gallery from a SMLM biplane DNA-PAINT dataset in lateral (x-y) and axial (x-z) views. N = 3 independent repeats with similar results. **C**. Distribution of ring diameter r = 108.9 ± 2.7 nm (mean ± standard deviation, 82 NPCs from one MINFLUX 3D dataset) and ring spacing s = 47.2 ± 2.7 nm (mean ± standard deviation, 82 NPCs from one MINFLUX 3D dataset) obtained from MINFLUX data. **D**. Distribution of ring diameter r = 114.9 ± 3.4 nm (mean ± standard deviation, 87 NPCs from one SMLM biplane 3D dataset) and ring spacing s = 53.4 ± 6.8 nm (mean ± standard deviation, 87 NPCs from one SMLM biplane 3D dataset) from SMLM data. **E**. Comparison of mean ring diameters and ring spacings measured with MINFLUX or SMLM images; MINFLUX derived mean ring diameter r = 111.9 ± 2.2 nm (mean ± standard deviation, 5 MINFLUX datasets from N = 2 independent realizations, 365 NPCs in total) vs SMLM mean ring diameter r = 117.9 ± 4.1 nm (mean ± standard deviation, 4 SMLM datasets from N = 2 independent realizations, 228 NPCs in total); MINFLUX derived mean ring spacing s = 49.6 ± 1.9 nm (mean ± standard deviation, 5 MINFLUX datasets from N = 2 independent realizations, 365 NPCs in total) vs SMLM mean ring spacing s = 58.9 ± 3.9 nm (mean ± standard deviation, 4 SMLM datasets from N = 2 independent realizations, 228 NPCs in total). **F**. Averaged 3D NPC Nup96 images from an analyzed MINFLUX dataset (top row, N = 29 NPCs, 1396 combined localizations) versus an averaged 3D NPC Nup96 image from a SMLM dataset (bottom row, N = 72 NPCs, 4512 combined localizations). Lateral views of the nucleoplasmic side (x-y) and axial side-views (x-z) are shown. ~12nm distal peaks resulting from adjacent Nup96 sites are indicated in the MINFLUX nucleoplasmic view (arrows). Box plots show the median (center line), the 25th and 75th percentiles (bounds of the box), and the mean (large circular marker). Whiskers extend to the most extreme data points within 1.5x the interquartile range, and all individual data points are displayed as a swarm overlay. Scale bars: 50 nm. Source data are provided with this paper.

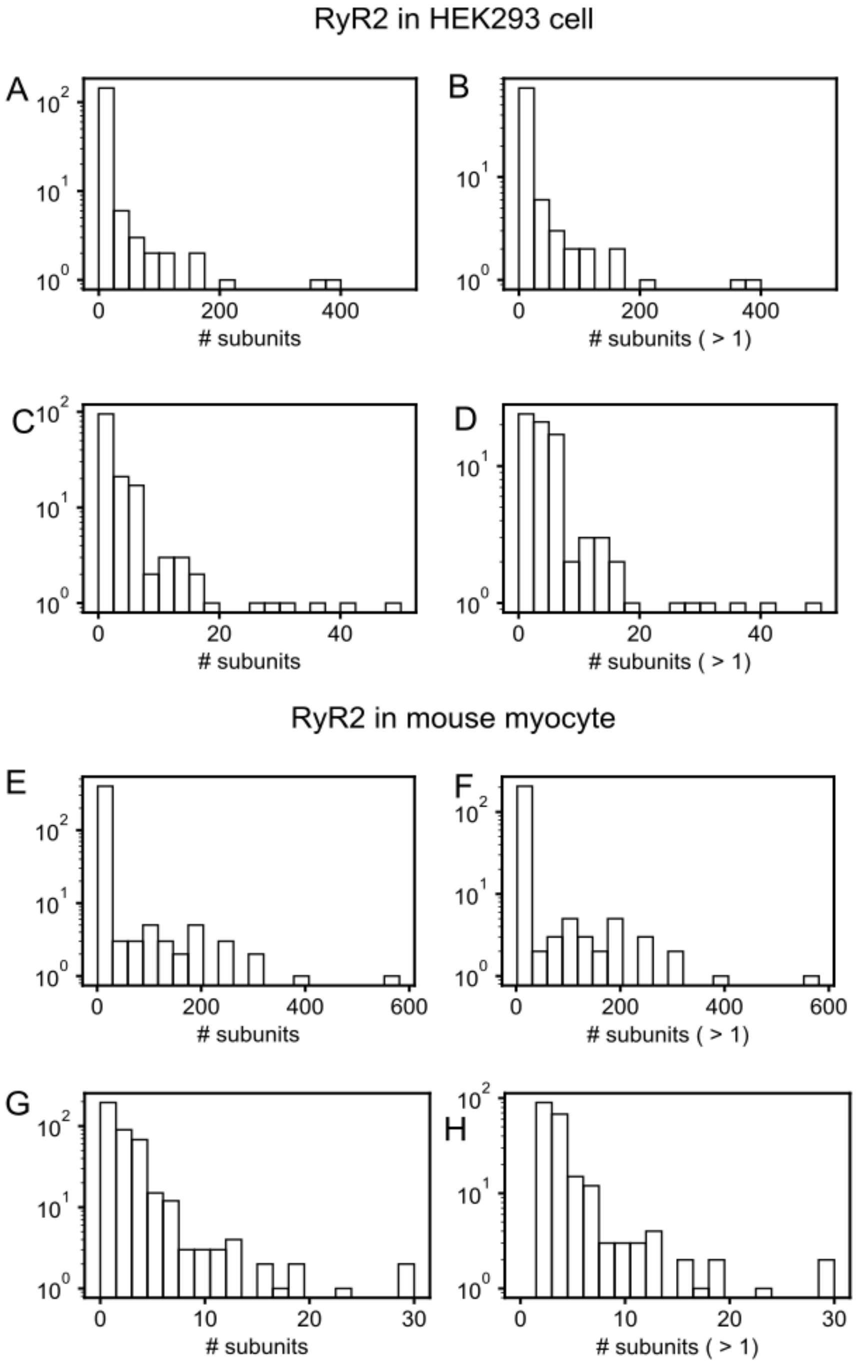

Supplementary Figure 6. Cluster size histograms. RyR2 cluster size histograms from a HEK293 cell (A-D) versus corresponding histograms from a mouse myocyte (E-H). Shown are detected subunit numbers using all clusters (left) and only including clusters with 2 or more subunits (right), highlighting the large number of isolated subunits, i.e. clusters containing a single or very few subunits. C, D and G, H show the same data as the corresponding panels above, respectively, but focusing on small clusters omitting the tail of large clusters that are prominent in visualizations of the data. Note the logarithmic display of cluster numbers. Source data are provided with this paper.

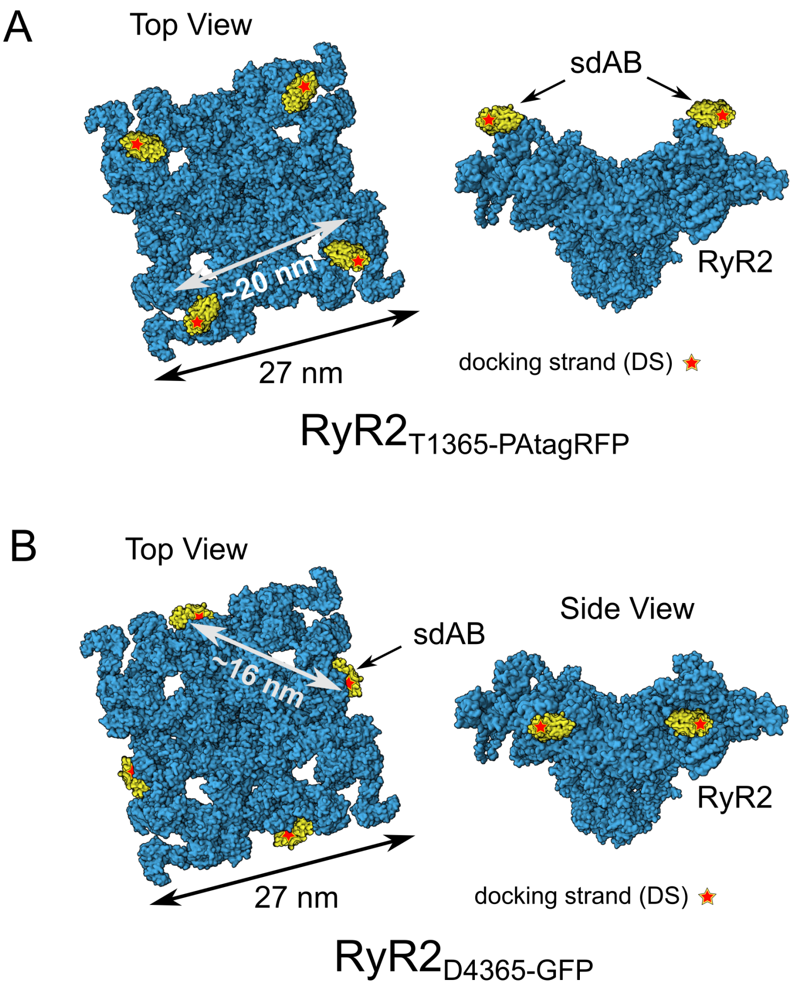

Supplementary Figure 7. RyR2 labeling schematics. **A**. RyR2$_{T1365\text{-}PATagRFP}$. The location of PA-TagRFP inserts and bound MP anti-TagFP used in PA-RFP RyR2 knock-in mice for both top-down (left) and side views (right). Inserts are situated close to the four corners and are spaced ~20 nm between vertices. **B**. RyR2$_{D4365\text{-}GFP}$. The location of GFP residues on RyR2 tetramers and the corresponding single-domain antibody location in HEK293 cells are expressed close to the midpoint of the edges of the RyR2 tetramer with nearest distances between GFP molecules of ~16 nm. Position of sdAB markers are shown based on GFP domain locations read out from figures in references[24,25].

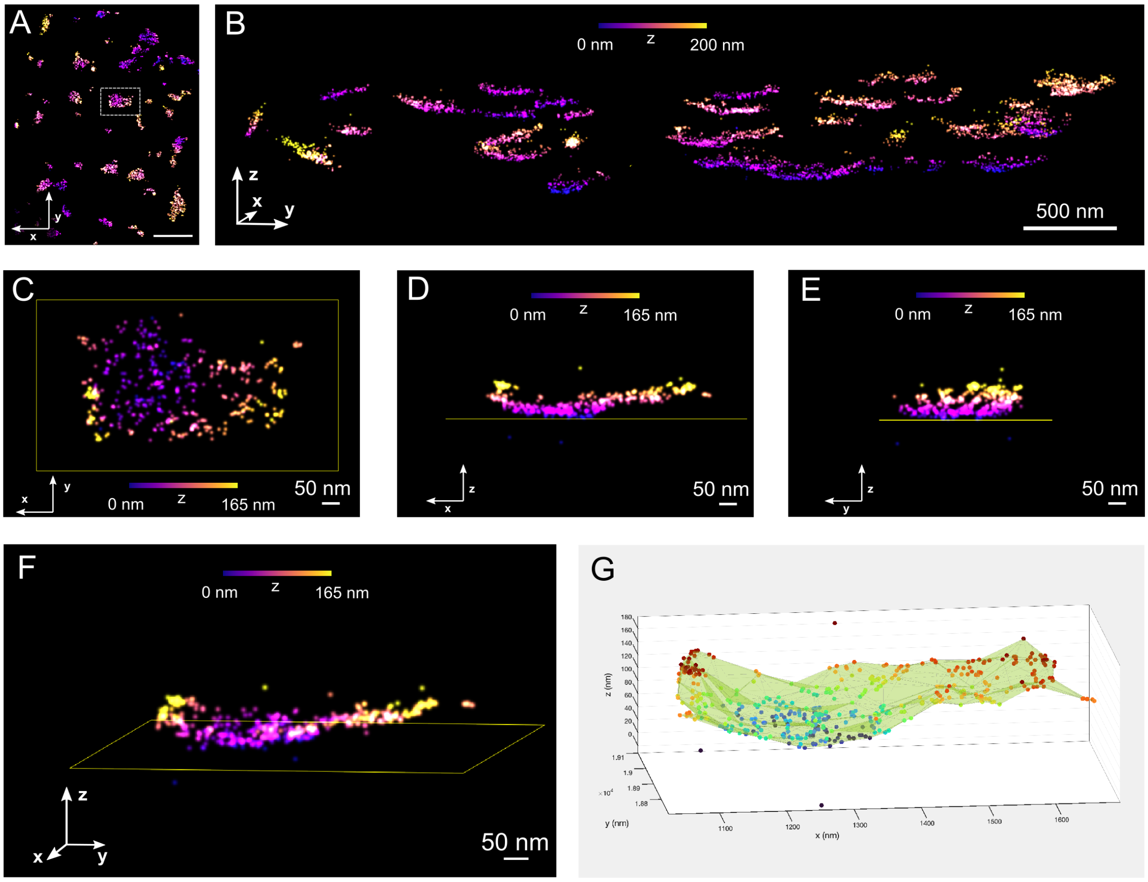

Supplementary Figure 8. Cluster 3D shapes as seen in in 3D views of MINFLUX 3D data in a mouse ventricular myocyte from a tagRFP RyR2 mouse. **A**. x-y overview of a typical dataset. N = 5 independent repeats with similar results. **B**. 3D view of all large clusters (containing >50 subunits). **C-F**. 3D views of a single cluster exhibiting a shape typically observed, i.e. curving gently away from the coverslip surface. The standard deviation of z locations in this cluster is 27 nm. **G**. Analysis of alpha-shape volume of the cluster shown in C-F, visualized using the MATLAB alphaShape functionality with default parameters. Outliers in the z position were detected as values that are more than three scaled median absolute deviations from the median position, using the MATLAB isoutlier function. A-G: color indicates z elevation. Scale bar in A: 1 µm, other panels as indicated.

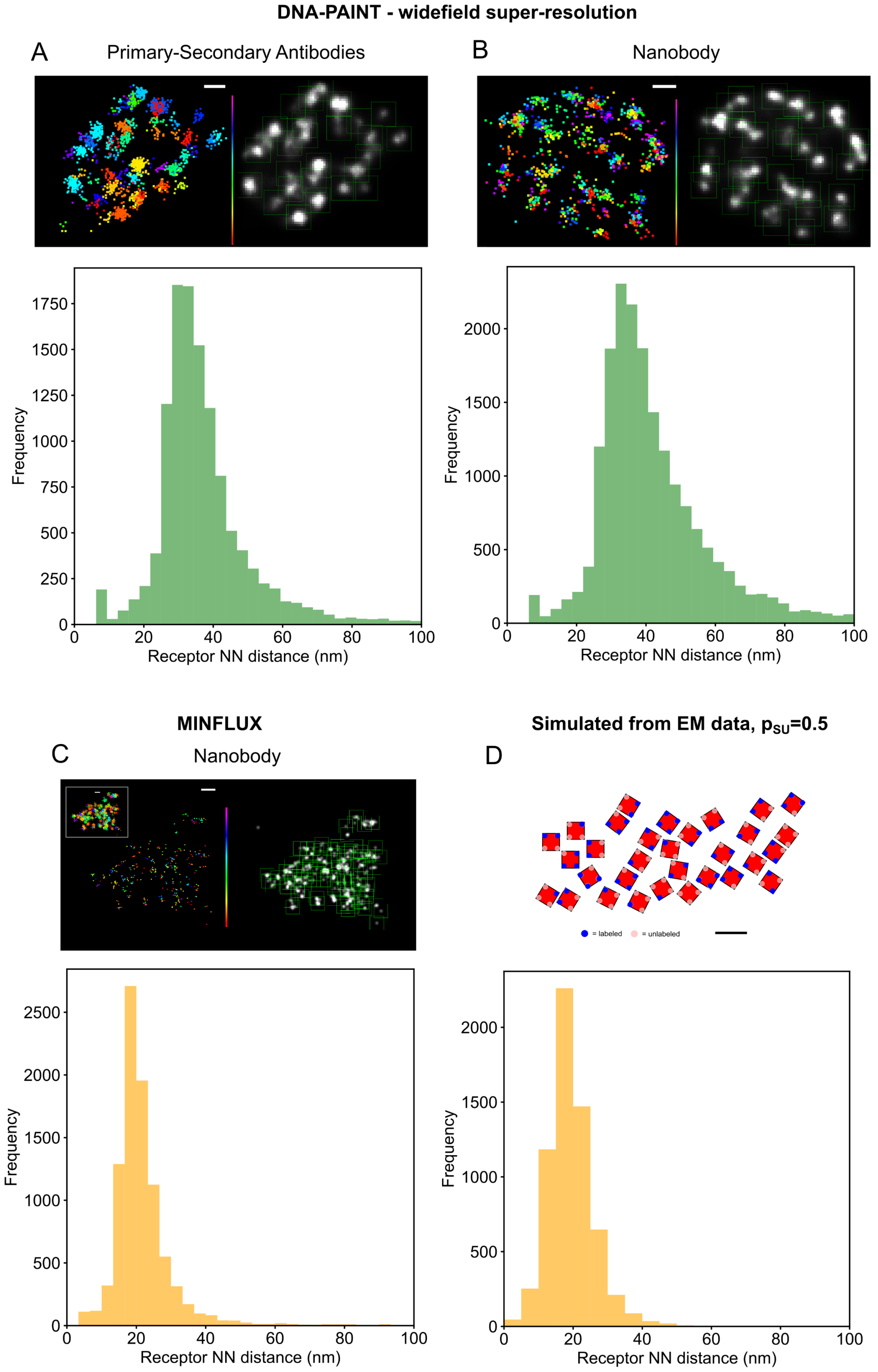

Supplementary Figure 9. Nearest neighbor (NN) distance analysis of RyR2 staining blobs in different staining and super-resolution imaging modalities in peripheral couplings of isolated mouse cardiomyocytes. Example localization data of RyR2 clusters are color coded for when in time the events were detected and accompanying rendered images with blob detection boxes (green) overlayed, see methods. The resulting nearest neighbor histograms from using blob centroid locations show similar distributions between labeling approaches in widefield super-resolution data but differ between imaging modalities. **A**. DNA-PAINT data for RyR2s labeled with primary & secondary antibodies had a median value of 34.2 nm and mode at 30 nm (N = 5 cells from n = 2 animals) compared to **B**, a median of 38.1 nm and mode at 35 nm (N = 9 cells from n = 3 animals) for single-domain antibodies, which are not statistically different (p = 0.31). **C**. The MINFLUX DNA-PAINT data, which also used the single-domain antibody, had a median nearest neighbor distance of 20.0 nm (smaller than widefield DNA-PAINT based imaging measures with ABs or sdABs, p < 0.001, respectively) and modal distance of 20 nm (N = 5 cells from n = 3 animals). The inset in the top image shows localizations before coalescing localizations with the same trace ID. The larger image of localizations shows the resulting coalesced localizations. **D**. Comparison simulation data generated from EM tomography data, references[22,23], with a labeling efficiency of 0.5 and localization error of 3.5 nm, resulted in a distribution very similar to the MINFLUX data, with median value 18.6 nm and mode at 17 nm. Scale bars A-C: 50 nm, D: 100 nm. Source data are provided with this paper.

References

1. Schmidt, R., Weihs, T., Wurm, C. A., Jansen, I., Rehman, J., Sahl, S. J. & Hell, S. W. MINFLUX nanometer-scale 3D imaging and microsecond-range tracking on a common fluorescence microscope. *Nat. Commun.* **12**, 1478 (2021).
2. Marin, Z., Graff, M., Barentine, A. E. S., Soeller, C., Chung, K. K. H., Fuentes, L. A. & Baddeley, D. PYMEVisualize: an open-source tool for exploring 3D super-resolution data. *Nat. Methods* **18**, 582–584 (2021).
3. Gwosch, K. C., Pape, J. K., Balzarotti, F., Hoess, P., Ellenberg, J., Ries, J. & Hell, S. W. MINFLUX nanoscopy delivers 3D multicolor nanometer resolution in cells. *Nat. Methods* **17**, 217–224 (2020).
4. Lin, R., Clowsley, A. H., Lutz, T., Baddeley, D. & Soeller, C. 3D super-resolution microscopy performance and quantitative analysis assessment using DNA-PAINT and DNA origami test samples. *Methods* **174**, 56–71 (2019).
5. Ostersehlt, L. M., Jans, D. C., Wittek, A., Keller-Findeisen, J., Inamdar, K., Sahl, S. J., Hell, S. W. & Jakobs, S. DNA-PAINT MINFLUX nanoscopy. *Nat. Methods* **19**, 1072–1075 (2022).
6. Pape, J. K., Stephan, T., Balzarotti, F., Büchner, R., Lange, F., Riedel, D., Jakobs, S. & Hell, S. W. Multicolor 3D MINFLUX nanoscopy of mitochondrial MICOS proteins. *Proc. Natl. Acad. Sci.* **117**, 20607–20614 (2020).
7. Nieuwenhuizen, R. P. J., Lidke, K. A., Bates, M., Puig, D. L., Grünwald, D., Stallinga, S. & Rieger, B. Measuring image resolution in optical nanoscopy. *Nat. Methods* **10**, 557–562 (2013).
8. Banterle, N., Bui, K. H., Lemke, E. A. & Beck, M. Fourier ring correlation as a resolution criterion for super-resolution microscopy. *J. Struct. Biol.* **183**, 363–367 (2013).
9. Thevathasan, J. V., Kahnwald, M., Cieśliński, K., Hoess, P., Peneti, S. K., Reitberger, M., Heid, D., Kasuba, K. C., Hoerner, S. J., Li, Y., Wu, Y.-L., Mund, M., Matti, U., Pereira, P. M., Henriques, R., Nijmeijer, B., Kueblbeck, M., Sabinina, V. J., Ellenberg, J. & Ries, J. Nuclear pores as versatile reference standards for quantitative superresolution microscopy. *Nat. Methods* **16**, 1045–1053 (2019).
10. Schindelin, J., Arganda-Carreras, I., Frise, E., Kaynig, V., Longair, M., Pietzsch, T., Preibisch, S., Rueden, C., Saalfeld, S., Schmid, B., Tinevez, J.-Y., White, D. J., Hartenstein, V., Eliceiri, K.,

Tomancak, P. & Cardona, A. Fiji: an open-source platform for biological-image analysis. *Nat. Methods* **9**, 676–682 (2012).

11. Wu, Y.-L., Hoess, P., Tschanz, A., Matti, U., Mund, M. & Ries, J. Maximum-likelihood model fitting for quantitative analysis of SMLM data. *Nat. Methods* **20**, 139–148 (2023).

12. Wales, D. J. & Doye, J. P. K. Global Optimization by Basin-Hopping and the Lowest Energy Structures of Lennard-Jones Clusters Containing up to 110 Atoms. *J. Phys. Chem. A* **101**, 5111–5116 (1997).

13. Zhu, C., Byrd, R. H., Lu, P. & Nocedal, J. Algorithm 778: L-BFGS-B: Fortran subroutines for large-scale bound-constrained optimization. *ACM Trans Math Softw* **23**, 550–560 (1997).

14. Sau, A., Schnorrenberg, S., Huang, Z., Bandyopadhyay, D., Sharma, A., Gürth, C.-M., Dave, S. & Musser, S. M. Overlapping nuclear import and export paths unveiled by two-colour MINFLUX. *Nature* **640**, 821–827 (2025).

15. Zimmerli, C. E., Allegretti, M., Rantos, V., Goetz, S. K., Obarska-Kosinska, A., Zagoriy, I., Halavatyi, A., Hummer, G., Mahamid, J., Kosinski, J. & Beck, M. Nuclear pores dilate and constrict in cellulo. *Science* **374**, eabd9776 (2021).

16. Dasgupta, A., Deschamps, J., Matti, U., Hübner, U., Becker, J., Strauss, S., Jungmann, R., Heintzmann, R. & Ries, J. Direct supercritical angle localization microscopy for nanometer 3D superresolution. *Nat. Commun.* **12**, 1180 (2021).

17. Wang, J., Allgeyer, E. S., Sirinakis, G., Zhang, Y., Hu, K., Lessard, M. D., Li, Y., Diekmann, R., Phillips, M. A., Dobbie, I. M., Ries, J., Booth, M. J. & Bewersdorf, J. Implementation of a 4Pi-SMS super-resolution microscope. *Nat. Protoc.* **16**, 677–727 (2021).

18. Sobie, E. A., Guatimosim, S., Gómez-Viquez, L., Song, L.-S., Hartmann, H., Jafri, M. S. & Lederer, W. J. The Ca2+ leak paradox and rogue ryanodine receptors: SR Ca2+ efflux theory and practice. *Prog. Biophys. Mol. Biol.* **90**, 172–185 (2006).

19. Baddeley, D., Jayasinghe, I. D., Lam, L., Rossberger, S., Cannell, M. B. & Soeller, C. Optical single-channel resolution imaging of the ryanodine receptor distribution in rat cardiac myocytes. *Proc. Natl. Acad. Sci. U. S. A.* **106**, 22275–22280 (2009).

20. Edelsbrunner, H., Kirkpatrick, D. & Seidel, R. On the shape of a set of points in the plane. *IEEE Trans. Inf. Theory* **29**, 551–559 (1983).

21. Edelsbrunner, H. & Mücke, E. P. Three-dimensional alpha shapes. *ACM Trans Graph* **13**, 43–72 (1994).

22. Asghari, P., Scriven, D. R. L., Sanatani, S., Gandhi, S. K., Campbell, A. I. M. & Moore, E. D. W. Nonuniform and variable arrangements of ryanodine receptors within mammalian ventricular couplons. *Circ. Res.* **115**, 252–262 (2014).

23. Asghari, P., Scriven, D. R., Ng, M., Panwar, P., Chou, K. C., van Petegem, F. & Moore, E. D. Cardiac ryanodine receptor distribution is dynamic and changed by auxiliary proteins and post-translational modification. *eLife* **9**, e51602 (2020).

24. Liu, Z., Zhang, J., Li, P., Chen, S. R. W. & Wagenknecht, T. Three-dimensional Reconstruction of the Recombinant Type 2 Ryanodine Receptor and Localization of Its Divergent Region 1*. *J. Biol. Chem.* **277**, 46712–46719 (2002).

25. Liu, Z., Zhang, J., Wang, R., Wayne Chen, S. R. & Wagenknecht, T. Location of Divergent Region 2 on the Three-dimensional Structure of Cardiac Muscle Ryanodine Receptor/Calcium Release Channel. *J. Mol. Biol.* **338**, 533–545 (2004).

26. Sehnal, D., Bittrich, S., Deshpande, M., Svobodová, R., Berka, K., Bazgier, V., Velankar, S., Burley, S. K., Koča, J. & Rose, A. S. Mol* Viewer: modern web app for 3D visualization and analysis of large biomolecular structures. *Nucleic Acids Res.* **49**, W431–W437 (2021).

27. Soeller, C. csoeller/PYME-test-env: PYME-test-env release Nov 2025: version v1.0.4. Zenodo https://doi.org/10.5281/zenodo.17768735 (2025).

28. Baddeley, D., Barentine, A. E. S., Marin, Z., Soeller, Christian, Yujin-Bao, actions-user, Chung, K., adrianhollow, Pinto, D. M. S., Mick, Balaji, A., & Akalanka. csoeller/python-microscopy: python-microscopy 25.05.16: version 25.05.16. Zenodo https://doi.org/10.5281/zenodo.17768283 (2025).

29. Soeller, C. & Clowsley, A. csoeller/PYME-extra: PYME-extra-25.11.29-release: version 25.11.29. Zenodo https://doi.org/10.5281/zenodo.17763621 (2025).

30. Soeller, C., Clowsley, A. H. & Meletiou, A. MINFLUX RyR2 imaging data - analysis and simulation scripts. figshare https://doi.org/10.6084/m9.figshare.23733579.v3 (2025).

31. Soeller, C. & Clowsley, A. MINFLUX RyR2 DNA-PAINT Raw Data. figshare https://doi.org/10.6084/m9.figshare.29196023.v2 (2025).
