# Supplementary material for: MINFLUX microscopy resolves subunits of the cardiac ryanodine receptor and its 3D orientation in cells
Source: Nat Commun. 2025 Dec 21;17:1044. doi: 10.1038/s41467-025-67801-6 (PMC12847994; doi:10.1038/s41467-025-67801-6)
Supplement: Supplementary file 2 — Reporting Summary [file 41467_2025_67801_MOESM2_ESM.pdf]

nature portfolio

Corresponding author(s): Christian Soeller

Last updated by author(s): Dec 5, 2025

Reporting Summary

Nature Portfolio wishes to improve the reproducibility of the work that we publish. This form provides structure for consistency and transparency in reporting. For further information on Nature Portfolio policies, see our Editorial Policies and the Editorial Policy Checklist.

Statistics

For all statistical analyses, confirm that the following items are present in the figure legend, table legend, main text, or Methods section.

n/a	Confirmed	
☐	☒	The exact sample size (*n*) for each experimental group/condition, given as a discrete number and unit of measurement
☐	☒	A statement on whether measurements were taken from distinct samples or whether the same sample was measured repeatedly
☐	☒	The statistical test(s) used AND whether they are one- or two-sided<br>*Only common tests should be described solely by name; describe more complex techniques in the Methods section.*
☒	☐	A description of all covariates tested
☐	☒	A description of any assumptions or corrections, such as tests of normality and adjustment for multiple comparisons
☐	☒	A full description of the statistical parameters including central tendency (e.g. means) or other basic estimates (e.g. regression coefficient) AND variation (e.g. standard deviation) or associated estimates of uncertainty (e.g. confidence intervals)
☐	☒	For null hypothesis testing, the test statistic (e.g. *F*, *t*, *r*) with confidence intervals, effect sizes, degrees of freedom and *P* value noted<br>*Give P values as exact values whenever suitable.*
☒	☐	For Bayesian analysis, information on the choice of priors and Markov chain Monte Carlo settings
☒	☐	For hierarchical and complex designs, identification of the appropriate level for tests and full reporting of outcomes
☒	☐	Estimates of effect sizes (e.g. Cohen's *d*, Pearson's *r*), indicating how they were calculated

Our web collection on statistics for biologists contains articles on many of the points above.

Software and code

Policy information about availability of computer code

Data collection

Data was collected using Abberior Imspector version 16.3 MINFLUX on an Abberior MINFLUX microscope. Additional widefield SMLM data was collected using the open-source Python Microscopy Environment (PyME) on a modified Nikon Eclipse Ti-E inverted microscope with biplane used for 3D detections.

Data analysis

All source data and analysis scripts have been made available through a source file (available with the paper) and a figshare repository at the DOI https://doi.org/10.6084/m9.figshare.23733579. The following software versions were used and made available through zenodo DOIs:

PYME-test-env
https://github.com/csoeller/PYME-test-env - installer scripts to build environment
Release version: v1.0.4
Zenodo DOI: https://doi.org/10.5281/zenodo.17768735

python-microscopy
https://github.com/csoeller/python-microscopy - python package for microscopy
Release version: 25.05.16
Zenodo DOI: https://doi.org/10.5281/zenodo.17768283

PYME-extra
https://github.com/csoeller/PYME-extra - plugins for python-microscopy
Release version: 25.11.29

Zenodo DOI: https://doi.org/10.5281/zenodo.17763621

For manuscripts utilizing custom algorithms or software that are central to the research but not yet described in published literature, software must be made available to editors and reviewers. We strongly encourage code deposition in a community repository (e.g. GitHub). See the Nature Portfolio guidelines for submitting code & software for further information.

Data

Policy information about availability of data

All manuscripts must include a data availability statement. This statement should provide the following information, where applicable:

- Accession codes, unique identifiers, or web links for publicly available datasets
- A description of any restrictions on data availability
- For clinical datasets or third party data, please ensure that the statement adheres to our policy

All source data and analysis scripts have been made available through figshare at the DOI https://doi.org/10.6084/m9.figshare.23733579. Raw data is available in the related figshare repository at DOI https://doi.org/10.6084/m9.figshare.29196023. The source data underlying all graphs shown are provided as a Source Data file. Source data are provided with this paper.

Research involving human participants, their data, or biological material

Policy information about studies with human participants or human data. See also policy information about sex, gender (identity/presentation), and sexual orientation and race, ethnicity and racism.

Reporting on sex and gender	*Use the terms sex (biological attribute) and gender (shaped by social and cultural circumstances) carefully in order to avoid confusing both terms. Indicate if findings apply to only one sex or gender; describe whether sex and gender were considered in study design; whether sex and/or gender was determined based on self-reporting or assigned and methods used. Provide in the source data disaggregated sex and gender data, where this information has been collected, and if consent has been obtained for sharing of individual-level data; provide overall numbers in this Reporting Summary. Please state if this information has not been collected. Report sex- and gender-based analyses where performed, justify reasons for lack of sex- and gender-based analysis.*
Reporting on race, ethnicity, or other socially relevant groupings	*Please specify the socially constructed or socially relevant categorization variable(s) used in your manuscript and explain why they were used. Please note that such variables should not be used as proxies for other socially constructed/relevant variables (for example, race or ethnicity should not be used as a proxy for socioeconomic status). Provide clear definitions of the relevant terms used, how they were provided (by the participants/respondents, the researchers, or third parties), and the method(s) used to classify people into the different categories (e.g. self-report, census or administrative data, social media data, etc.) Please provide details about how you controlled for confounding variables in your analyses.*
Population characteristics	*Describe the covariate-relevant population characteristics of the human research participants (e.g. age, genotypic information, past and current diagnosis and treatment categories). If you filled out the behavioural & social sciences study design questions and have nothing to add here, write "See above."*
Recruitment	*Describe how participants were recruited. Outline any potential self-selection bias or other biases that may be present and how these are likely to impact results.*
Ethics oversight	*Identify the organization(s) that approved the study protocol.*

Note that full information on the approval of the study protocol must also be provided in the manuscript.

Field-specific reporting

Please select the one below that is the best fit for your research. If you are not sure, read the appropriate sections before making your selection.

[x] Life sciences [ ] Behavioural & social sciences [ ] Ecological, evolutionary & environmental sciences

For a reference copy of the document with all sections, see nature.com/documents/nr-reporting-summary-flat.pdf

Life sciences study design

All studies must disclose on these points even when the disclosure is negative.

Sample size	RyR subunit distribution in HEK293 cells and NPC measurements in U-2 OS Nup96-mEGFP cells were determined from N=10 MINFLUX datasets f(from N=22 cells) rom n=3 independent biological repeats. In cardiomyocytes N=22 MINFLUX datasets (from N=22 different cells) from 5 biological repeats (using 5 different animals) were obtained and analyzed. The primary focus of this study was not on statistically significant differences between different treatments/samples, therefore no statistical power analysis could be meaningfully conducted. The primary focus was on replicates to obtain representative mean quantities which led us to obtain >= 3 independent biological repeats.
Data exclusions	No data was excluded.
Replication	Experiments were conducted with >= 3 independent repeats (in the case of cardiomyocytes using data from 5 animals; in the case of HEK293 cells using 3 independent biological replicates which involved growing cells on coverslips, staining and imaging on different occasions in

	different weeks). The findings were generally replicated as indicated by the boxplots in figures 4 and 5. Detailed information is provided in the relevant figure legends.
Randomization	Cardiomyocytes from mice expressing PA-TagRFP on the RyR were randomly selected for MINFLUX imaging of RyR localization. Similarly, cells imaged in a monolayer of HEK293 stably expressing RyR2D4365-GFP that were in close proximity to U-2 OS Nup96-mEGFP cells were randomly selected. No experimental groups were used, therefore assignment to groups was not performed.
Blinding	In these experiments, analysis was performed on a single type of cardiomyocyte (ie. those from healthy mice expressing PA-TagRFP on the RyR) and on co-cultured cells (HEK293 stably expressing RyR2D4365-GFP and U-2 OS Nup96-mEGFP) and thus blinding to a treatment/ assignement to an experimental group was not applicable.

Reporting for specific materials, systems and methods

We require information from authors about some types of materials, experimental systems and methods used in many studies. Here, indicate whether each material, system or method listed is relevant to your study. If you are not sure if a list item applies to your research, read the appropriate section before selecting a response.

Materials & experimental systems

n/a	Involved in the study
☐	☒ Antibodies
☐	☒ Eukaryotic cell lines
☒	☐ Palaeontology and archaeology
☐	☒ Animals and other organisms
☒	☐ Clinical data
☒	☐ Dual use research of concern
☒	☐ Plants

Methods

n/a	Involved in the study
☒	☐ ChIP-seq
☒	☐ Flow cytometry
☒	☐ MRI-based neuroimaging

Antibodies

Antibodies used	Most experiments used Massive-Tag-Q anti-TagFP single domain antibody (Massive-Photonics, clone 1H7, no batch N°) (against tagFPs including tagRFP), TAG-X2 anti-GFP from the same manufacturer (clones 1H1 and 1B2, batch N°3302002), or TAG-X2-FAST anti-GFP (clones 1H1 and 1B2, no batch N°) . These nanobodies had either the docking strand DS3 utilizing the Imager 3 or the docking strand F3 ("Fast"), utilizing the Imager F3 ("Fast"), all with undisclosed sequences; some experiments used a primary anti-RyR antibody (MA3-916, C3-33, Lot N° XC345381, Thermo Fisher Scientific, Waltham, MA, USA) and secondary goat anti-mouse IgG antibodies (Jackson ImmunoResearch).
Validation	TAG-X2 anti-GFP was verified by NanoTag, for its specificity to GFP and its derivatives according to a specificity chart available at https://nano-tag.com/wp-content/uploads/2022/05/FP-chart-1.pdf. No non-specific staining was observed in cells lacking GFP. Massive-Tag-Q anti-TagFP was similarly verified by NanoTag, no non-specific staining was observed in cells lacking tagRFP.<br><br>According to the manufacturer, the primary antibody MA3-916 was verified by relative expression to ensure that the antibody binds to the antigen stated (https://www.thermofisher.com/antibody/product/Ryanodine-Receptor-Antibody-clone-C3-33-Monoclonal/MA3-916).

Eukaryotic cell lines

Policy information about cell lines and Sex and Gender in Research

Cell line source(s)	HEK-293 cells stably expressing RyR2D4365-GFP; created in the laboratory of Dr. Wayne S.R. Chen and maintained in the laboratory of Pete Jones, University of Otago. U-2 OS-Nup96-mEGFP cells were purchased from Cytion (clone 195, #300174).
Authentication	Genotyping for HEK-293 was carried out by Pete Jones at the University of Otago. U-2 OS-Nup96-mEGFP were not further authenticated after purchase, except for verification of the expected GFP expression pattern at the nuclear pore.
Mycoplasma contamination	Tested negative
Commonly misidentified lines (See ICLAC register)	*Name any commonly misidentified cell lines used in the study and provide a rationale for their use.*

Animals and other research organisms

Policy information about studies involving animals; ARRIVE guidelines recommended for reporting animal research, and Sex and Gender in Research

Laboratory animals	This study was performed using genetically modified PA-RFP RyR2 mice on the C57BL/6/6N background that harbor PA-TagRFP inserted after T1365, within exon 31 of the mRyR2 gene on chromosome 13 as described in the methods of Hou, Y. et al. (2023) 'Live-

	cell photoactivated localization microscopy correlates nanoscale ryanodine receptor configuration to calcium sparks in cardiomyocytes', Nature Cardiovascular Research, 2(3), pp. 251–267. Housing and breeding were carried out in a departmental animal facility with controlled environmental conditions (22 °C, 40 % relative humidity, 12 hours light/dark cycle), with mice having free access to laboratory chow and water. In this study we used adult male mice (N = 5, 2 – 12 months old).
Wild animals	No wild animals were used in this study.
Reporting on sex	Experiments were performed on male mice. Sex was not considered for this study. The focus of this study was not on statistically significant differences between different treatments or sexes.
Field-collected samples	No field-collected samples were used in this study.
Ethics oversight	For experiments carried out at the University of Oslo, all animal protocols were performed in accordance with the Norwegian Animal Welfare Act and NIH Guidelines (NIH publication No. 85-23, revised 2011) and were approved by the Norwegian Food Safety Authority (permit number 8951). For experiments carried out at the University of Bern, all animal protocols were performed in accordance with the Swiss Animal Welfare Act and were approved by the Amt für Veterinärwesen of the Canton of Bern (permit number 35279).

Note that full information on the approval of the study protocol must also be provided in the manuscript.
