# Supplementary material for: MINFLUX microscopy resolves subunits of the cardiac ryanodine receptor and its 3D orientation in cells
Source: Nat Commun. 2025 Dec 21;17:1044. doi: 10.1038/s41467-025-67801-6 (PMC12847994; doi:10.1038/s41467-025-67801-6)
Supplement: Supplementary file 3 — Description of Additional Supplementary Files [file 41467_2025_67801_MOESM3_ESM.pdf]

Supplementary Movie 1:
3D MINFLUX localization data of a HEK293 cell expressing the RyR2-GFP fusion protein RyR2D4365-GFP visualized in the PyME software.

Supplementary Movie 2:
3D MINFLUX localization data of an isolated PA-RFP knock-in mouse cardiomyocyte expressing RyR2T1365-PATagRFP visualized in the PyME software.
